# Expression patterns of m^6^A RNA methylation regulators under apoptotic conditions in various human cancer cell lines

**DOI:** 10.55730/1300-0152.2679

**Published:** 2023-12-14

**Authors:** Azime AKÇAÖZ ALASAR, Buket SAĞLAM, İpek ERDOĞAN VATANSEVER, Bünyamin AKGÜL

**Affiliations:** Department of Molecular Biology and Genetics, Noncoding RNA Laboratory, İzmir Institute of Technology, İzmir, Turkiye

**Keywords:** Epitranscriptomics, m^6^A modification, cancer, apoptosis, cisplatin, TNF-α

## Abstract

**Background/aim:**

Cancer is a complex disease that involves both genetic and epigenetic factors. While emerging evidence clearly suggests that changes in epitranscriptomics play a crucial role in cancer pathogenesis, a comprehensive understanding of the writers, erasers, and readers of epitranscriptomic processes, particularly under apoptotic conditions remains lacking. The aim of this study was to uncover the changes in the expression of m^6^A RNA modifiers under apoptotic conditions across various cancer cell lines.

**Materials and methods:**

Initially, we quantified the abundance of m^6^A RNA modifiers in cervical (HeLa and ME180), breast (MCF7 and MDA-MB-231), lung (A549 and H1299), and colon (Caco-2 and HCT116) cancer cell lines using qPCR. Subsequently, we induced apoptosis using cisplatin and tumor necrosis factor-alpha (TNF-α) to activate intrinsic and extrinsic pathways, respectively, and assessed apoptosis rates via flow cytometry. Further, we examined the transcript abundance of m^6^A RNA modifiers under apoptotic conditions in cervical, breast, and lung cancer cell lines using qPCR.

**Results:**

Overall, treatment with cisplatin increased the abundance of m^6^A modifiers, whereas TNF-α treatment decreased their expression in cervical, breast, and lung cancer cell lines. Specifically, cisplatin-induced apoptosis, but not TNF-α-mediated apoptosis, resulted in decreased abundance of METTL14 and FTO transcripts. Additionally, cisplatin treatment drastically reduced the abundance of IGF2BP2 and IGF2BP3 readers.

**Conclusion:**

These results suggest that the differential response of cancer cells to apoptotic inducers may be partially attributed to the expression of m^6^A RNA modifiers.

## 1. Introduction

Global cancer incidence in women, as reported by the World Health Organization, indicates that the most prevalent cancer types are breast, colorectal, lung, and cervical cancers, with frequencies of 25.8%, 9.9%, 8.8%, and 6.9%, respectively (Norpo’latovna, 2023; Siegel et al., 2023). Therefore, the ability to identify prognostic risk factors of cancer types and understand their growth, progression, and treatments has been highly valued by researchers. Although genetic, genomic, and biochemical approaches have unveiled numerous molecular mechanisms that contribute to tumorigenesis, recent developments have shown that N^6^-methyladenosine (m^6^A) RNA modification is closely associated with both activation and inhibition of tumorigenesis (Du et al., 2022; Ma et al., 2022; Zhang et al., 2022; Jiang et al., 2023). m^6^A RNA modification involves the attachment of a methyl group to the 6-carbon of adenosine, constituting a cotranscriptional and dynamically regulated mark catalyzed by a series of enzymes (Leonetti et al., 2020). Writers, erasers, and readers are responsible for the attachment, removal, and recognition of m^6^A sites, respectively, thereby modulating a wide range of RNA fates, including splicing, translation, microRNA processing, nuclear export, RNA stability, and decay (Niu et al., 2013; Akçaöz and Akgül, 2022). Consequently, m^6^A RNA methylation plays a pivotal role in orchestrating various biological processes such as gene regulation, DNA damage response, signal transduction, and apoptosis (Roundtree et al., 2017; Liu et al., 2018; Alasar et al., 2022; Akçaöz Alasar et al., 2024).

The existing evidence strongly indicates that certain m^6^A RNA writers, erasers, or readers are associated with cancer formation or development (Zhang et al., 2021; Deng et al., 2022; Liu et al., 2022). Much of the research has focused on Fat mass and obesity-associated protein (FTO) and Methyltransferase like 3 (METTL3). FTO functions as an oncogene in acute myeloid leukemia, promoting malignant transformation and tumor formation (Li et al., 2017). Similarly, upregulation of FTO mRNA and protein levels in human lung cancer tissues correlates with increased cell proliferation and tumor growth, while its downregulation leads to reduced proliferation and tumor growth (Li et al., 2019). On the other hand, decreased METTL3 levels impair tumor growth by reducing m^6^A methylation and inhibiting malignant transformation in glioblastoma (Visvanathan et al., 2018). However, proteins within the m^6^A machinery do not always act as oncogenes; in some cases, they may positively regulate tumor growth. For example, in gastric cancer with METTL3 overexpression, METTL3 knockdown hinders cell proliferation and migration capacity (Liu et al., 2019). Additionally, METTL3 induces tumorigenesis and growth in hepatocellular carcinoma (Chen et al., 2018). These results clearly suggest that the contribution of m^6^A machinery to carcinogenesis differs depending on the cancer type. Therefore, a comprehensive understanding of writer, eraser, and reader expression in this fundamental process is essential for deciphering the complex association between m^6^A RNA methylation and cancer. In this study, we assessed the abundance of transcripts involved in orchestrating m^6^A methylation in both healthy and various cancer cell lines, as well as their expression under apoptotic conditions.

## 2. Materials and methods

### 2.1. Cell Culture

HeLa cells were procured from DSMZ GmbH (Germany). ME-180 (HTB-33), MDA-MB-231 (HTB-26), and MCF-7 (HTB-22) cell lines were purchased from ATCC (United States). A549 (ATCC, CCL-185), Caco-2 (Republic of Türkiye Ministry of Agriculture and Forestry Foot and Mouth Disease Research Institute), H1299 (ATCC, HTB-37), and HCT116 (German Cancer Research Centre, Heidelberg, Germany) cell lines were kindly provided by Dr Serdar Özçelik of İzmir Institute of Technology (Türkiye), Dr. Sreeparna Banerjee of Middle East Technical University (Türkiye), Dr Hakan Akça of Pamukkale University (Türkiye), and Dr Semra Koçtürk of Dokuz Eylül University (Türkiye), respectively. Caco-2 cells were cultured in EMEM (Gibco, United States) supplemented with 20% FBS, 2 mM L-glutamine, 1 mM Na-pyruvate (Gibco, United States), and 1X nonessential amino acids (Gibco, United States). The other cell lines were maintained under the following conditions: RPMI 1640 (Gibco, United States) for HeLa and H1299 cells, DMEM (Lonza, Switzerland) with high glucose for A549 and MDA-MB-231, and McCoy’s 5A (Lonza, Switzerland) for ME-180 and HCT116 cells. All media, except for EMEM, were supplemented with 10% fetal bovine serum (FBS) (Gibco, United States) and 2 mM L-glutamine. HeLa, MCF7, and A549 cell lines were maintained in a humidified atmosphere of 5% CO_2_ at 37 °C. Treatments with 80 μM, 100 μM, and 80 μM of cisplatin (Santa Cruz Biotechnology, United States) for 16 h and 75 ng/mL TNF-α in 5 μg/mL cycloheximide (CHX) (Applichem, Germany), 10 ng/mL TNF-α in 5 μg/mL CHX, and 20 ng/mL TNF-α in 10 μg/mL CHX (Biolegend, United States) for 24 h treatments were performed using varying concentrations, respectively. For negative controls, 0.1% (v/v) DMSO and CHX were used for cisplatin and TNF-α treatments, respectively. Treated cells were harvested using 1X Trypsin-EDTA (Gibco, United States), washed in 1X cold PBS (Gibco, United States), and then stained with Annexin V-PE (Becton Dickinson, United States) and 7AAD (Becton Dickinson, United States) in the presence of 1X Annexin binding buffer (Becton Dickinson, United States) for flow cytometry analysis (Yaylak et al., 2019). Apoptotic and live populations were determined using a FACSCanto flow cytometer (Becton Dickinson, United States). All experiments were conducted in three biological replicates.

### 2.2. RNA isolation

Cells were lysed in an appropriate volume (750 μL for 5 × 10^6^ cells) of TRIzol reagent (Invitrogen, Thermo Fisher Scientific, Waltham, MA, USA) and stored at −80 °C until isolation. Total RNA isolation was performed following the manufacturer’s protocol. If necessary, 1 μL (20 mg/μL) of glycogen was added before precipitating total RNAs at +4 °C and 12,000 × *g* for 10 min. RNA pellets were air-dried for 5–10 min and then dissolved in 20–30 μL of nuclease-free water. The quantity and purity of RNA were measured using a NanoDrop ND-1000 UV-Vis Spectrophotometer (Thermo Scientific, Waltham, MA, USA), and RNAs were stored at −80 °C until use.

### 2.3. cDNA synthesis and qPCR

Total RNAs were converted to cDNA using the RevertAid first-strand cDNA synthesis kit (Thermo Fisher Scientific, United States) following the manufacturer’s instructions and diluted to 5 ng/μL equivalent of total RNAs with nuclease-free water for qPCR analysis. qPCR reactions were set up as follows: 6.25 μL of GoTaq qPCR Master Mix (Promega, Madison, WI, USA), 4.25 μL of nuclease-free water, 1 μL of 5 μM corresponding primer, and 1 μL of cDNA. The qPCR reactions were incubated at 95 °C for 2 min for initial denaturation, followed by 45 cycles of denaturation at 95 °C for 15 s and annealing at 60 °C for 1 min, with a melting step, in a Rotor-Gene Q machine (Qiagen, Hilden, Germany). All analyses were conducted in three biological replicates. GAPDH was used for normalization. Statistical analysis was performed using Student’s *t*-test, and p < 0.05 was considered statistically significant. Primers are listed in Table.

### 2.4. RNA-seq data analysis

RNA-seq data obtained from the three biological replicates of total RNAs of cisplatin-treated HeLa cells have been previously described (Gurer et al., 2021). We utilized the same methodology to analyze the RNA-seq data from doxorubicin-, TNF-α-, and FAS ligand-treated HeLa cells and used only the expression levels of RNA m^6^A modifiers as it is an unpublished data set.

## 3. Results

### 3.1. Abundance of transcripts of m^6^A writers and erasers is deregulated in cancer cells

**Figure 1:**
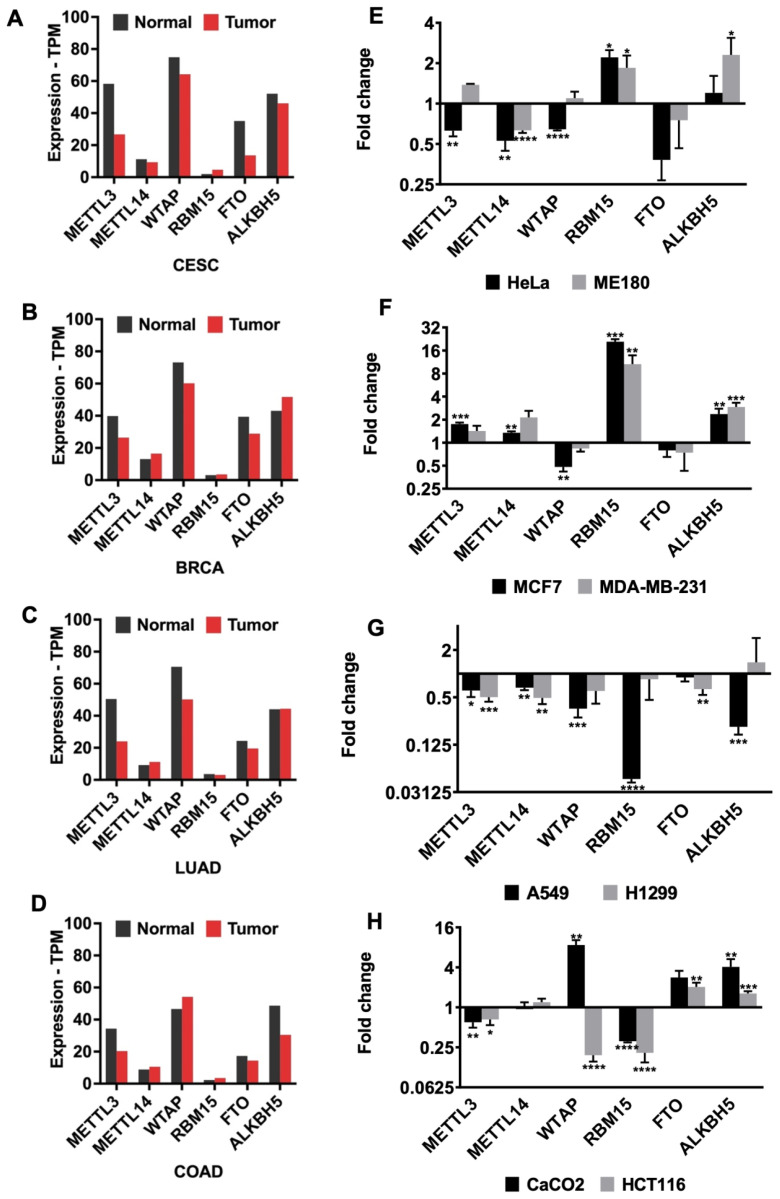
The gene expression profile of m^6^A regulators. **(A–D)** The gene expression level of writers and erasers across all tumor samples and paired normal tissues in CESC, BRCA, LUAD, and COAD. The expression analysis was performed using the GEPIA2 database. CESC: Cervical squamous cell carcinoma and endocervical adenocarcinoma; BRCA: Breast invasive carcinoma; LUAD: Lung adenocarcinoma; COAD: Colon adenocarcinoma. Gene expression levels of m^6^A writers (METTL3, METTL14, WTAP and RBM15) and erasers (FTO and ALKBH5) in **(E)** HeLa and ME180, **(F)** MCF7 and MDA-MB-231, **(G)** A549 and H1299 and **(H)** Caco-2 and HCT116 cancer cell lines. All cells were normalized with their healthy cell lines. All qPCR samples were normalized with GAPDH housekeeping gene. Error bars represent mean ± SD of three biological replicate samples (unpaired, two-tailed t-test). (*: p ≤ 0.05, **: p ≤ 0.01, ***: p ≤ 0.001, ****: p ≤ 0.0001)

The extent of m^6^A methylation is primarily determined by the dynamic action of writers and erasers (Li et al., 2023). Key components of the writer complex include METTL3, METTL14, WTAP, and RBM15, while FTO and ALKBH5 are involved in demethylation processes (Luo et al., 2023). Thus, we first examined the expression levels of METTL3, METTL14, WTAP, RBM15, FTO, and ALKBH5 transcripts in tumor samples and corresponding normal samples using the GEPIA database (Figures 1A–1D). Our analyses revealed lower abundance of METTL3 and FTO transcripts in cervical squamous cell carcinoma and endocervical adenocarcinoma (CESC), breast invasive carcinoma (BRCA), lung adenocarcinoma (LUAD), and colon adenocarcinoma (COAD) compared to their matched normal tissues. WTAP and ALKBH5 showed varied abundance across different cancer types analyzed (Figures 1A–1D). Subsequently, we examined the abundance of m^6^A regulators in total RNAs isolated from various cell lines, including healthy, nonmetastatic, and metastatic cells of cervical, breast, lung, and colon cancers. Specifically, we found lower expression of METTL3, METTL14, and WTAP transcripts, while RBM15 and ALKBH5 abundance was elevated in cervical cancer cells (Figure 1E). Interestingly, RBM15 was upregulated by 20-fold (p < 0.001) and 10-fold (p < 0.01) in nonmetastatic and metastatic breast cancer cells (MCF7 and MDA-MB-231), respectively, contrasting with indifferent expression in patient samples based on TCGA database data. TCGA data indicated very low and nearly equal levels of RBM15 expression in healthy and cancerous breast tissues, with 3 and 3.5 transcripts per million, respectively (Figures 1B and 1F). Additionally, ALKBH5 was upregulated by approximately 2.5-fold (p < 0.01) in breast cancer cells (Figure 1F). In lung cancer cell lines, all analyzed m^6^A regulators were downregulated to varying extents. In particular, the transcript abundance of RBM15 and ALKBH5 was reduced by 21-fold (p < 0.0001) and 4.7-fold (p < 0.001), respectively, in A549 cells (Figure 1G). The abundance of RBM15 and ALKBH5 in lung cancer cell lines exhibited a negative correlation with those in breast cancer cell lines. In colon cancer cell lines, METTL3 and RBM15 were downregulated, whereas transcript levels of FTO and ALKBH5 were upregulated (Figure 1H). We observed an 8.6-fold increase (p < 0.01) in WTAP RNA levels in nonmetastatic Caco-2 cells, whereas WTAP was downregulated by 5.2-fold (p < 0.0001) in metastatic colon cancer cell line HCT116 (Figure 1H).

### 3.2. Expression of m^6^A RNA methylation regulators under apoptotic conditions

**Figure 2:**
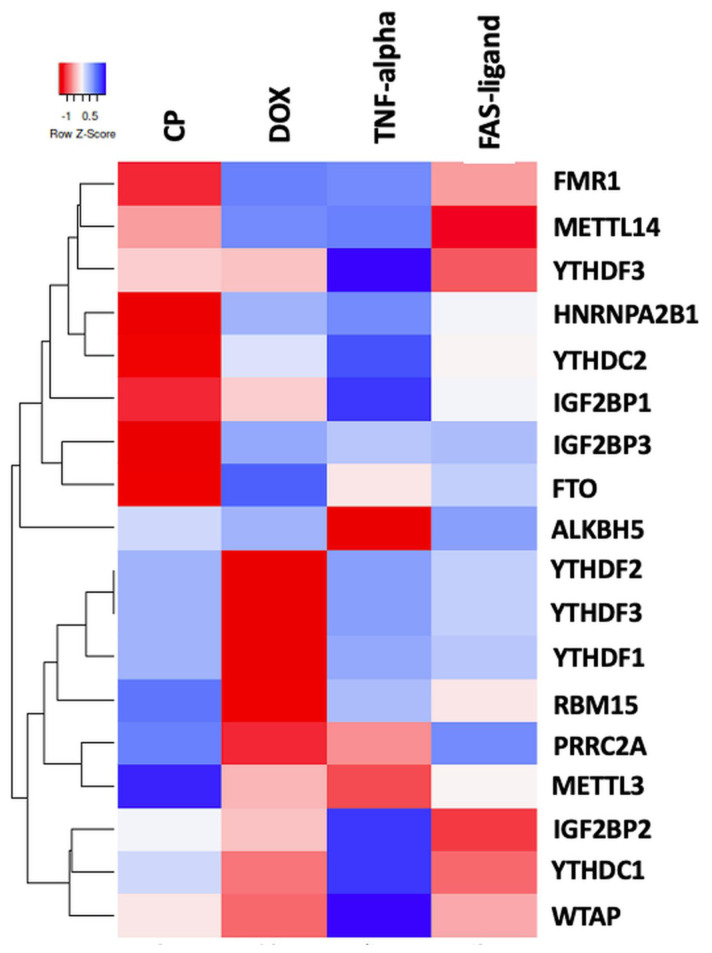
RNA-seq analysis for 4 drugs. Heatmap of differentially expressed m^6^A modifiers upon the induction of the extrinsic and intrinsic pathways of apoptosis. Heatmap of RNA-seq analysis for m^6^A related writer, eraser, and reader expressions after 4 drug treatments as CP, DOX, TNF-α, and Fas ligand. Annotations on the left part of the heatmap show clustering of the m^6^A-methylation-related expressions.

One of the important hallmarks of cancer is resistance to cell death and cancer cells are notorious for evading apoptotic pathways (Scheel and Schäfer, 2023). Cisplatin (CP) and doxorubicin (DOX) are widely used anticancer chemotherapy drugs that are widely used as inducers of the intrinsic apoptotic pathway (Motlagh et al., 2023).). Conversely, TNF-α and FAS ligands trigger the extrinsic apoptotic pathways by binding to their cell surface receptors (Xiao et al., 2023). To understand how the expression of m^6^A regulators is affected upon initiation of apoptosis in cancer cells, we analyzed RNA-seq data to examine the expression of m^6^A regulators in HeLa cells treated with cisplatin, doxorubicin, TNF-α, and FAS ligands. RNA sequencing data were obtained from HeLa cells treated with 80 μM CP for 16 h, 0.5 μM doxorubicin for 4 h, 0.5 μg/mL anti-Fas mAb for 16 h, and 125 ng/mL TNF-α for 8 h, resulting in an apoptosis rate of 50% in HeLa cells as previously reported (Yaylak et al., 2019). The cisplatin dataset has been published (Gurer et al., 2021), while the datasets for the other treatments are unpublished. Using these datasets, we analyzed the expression of a total of 18 m^6^A regulators (5 writers, 11 readers, and 2 erasers) under apoptotic conditions induced by cisplatin, doxorubicin, TNF-α, and FAS ligands. Interestingly, the expression levels of regulators displayed distinct patterns under all drug/ligand treatment conditions tested (Figure 2). Encouraged by the differential effects of intrinsic and extrinsic inducers of apoptosis, we utilized cisplatin as an inducer of the intrinsic pathway and TNF-α as an inducer of the extrinsic pathway to track the expression patterns of m^6^A RNA methylation regulators in different cancer cell lines.

### 3.3. Expression of m^6^A regulators is perturbed by cisplatin in different cancer cell lines

**Figure 3:**
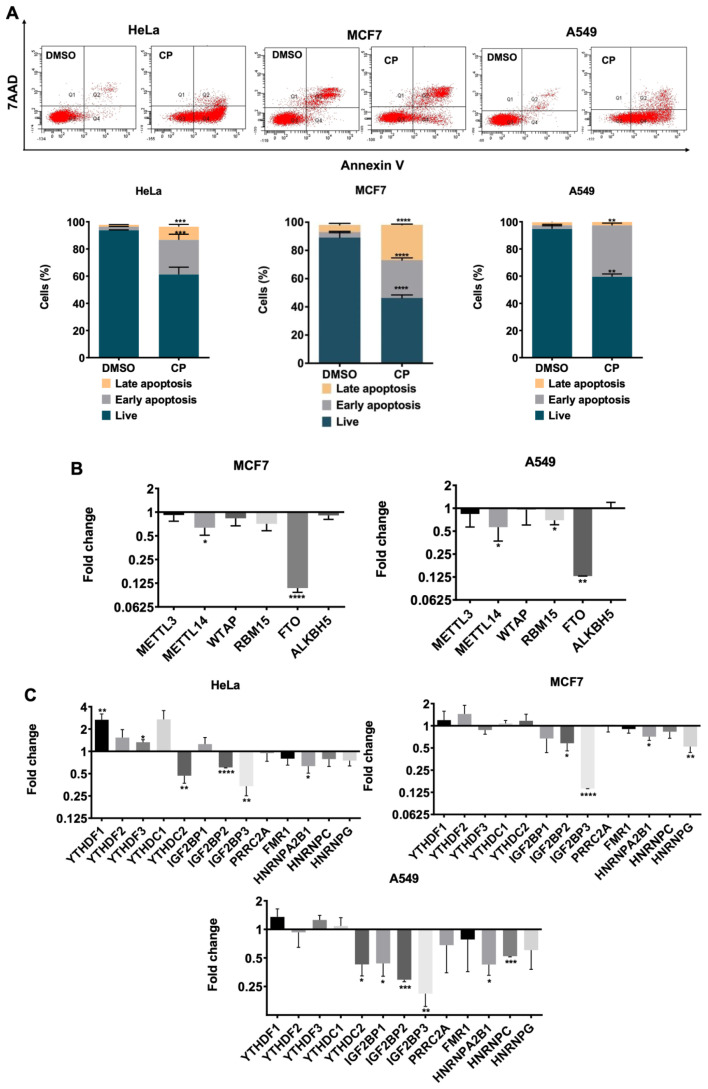
Gene expression analysis in cisplatin-induced apoptotic HeLa, MCF7, and A549 cells. **(A)** Relatively less metastatic HeLa, MCF7, and A549 cells were treated with 80 μM, 100 μM, and 80 μM of cisplatin for 16 h, respectively. 0.1% (v/v) DMSO was used as a control. **(B)** Gene expression levels of m^6^A writers (METTL3, METTL14, WTAP, and RBM15) and eraser genes (FTO and ALKBH5) in MCF7 (left) and A549 cells (right). **(C)** Gene expression levels of m^6^A readers in HeLa, MCF7, and A549 cells in CP treatment. Results were normalized against GAPDH. Experiments were conducted in three biological replicates. Data are presented as mean values ± SD. *: p ≤ 0.05, **: p≤0.01, ***: p ≤ 0.001, ****: p ≤ 0.0001 by a two-tailed unpaired *t*-test.

To uncover the expression pattern of m^6^A RNA methylation regulators under cisplatin-induced apoptotic conditions, we treated HeLa, MCF7, and A549 cells with varying concentrations of cisplatin. Previous reports indicated that 80 μM cisplatin induces approximately 50% early apoptosis in HeLa cells (Yaylak et al., 2019; Figure 3A). Cisplatin at a concentration of 100 μM induced 22.8% of early apoptosis as determined by Annexin V-positive early apoptotic cells in MCF7 cells (Figure 3A). A549 cells were subjected to 80 μM cisplatin for 24 h, which was sufficient to attain an early apoptotic rate of approximately 35% compared to the control group treated with 0.1% DMSO (Figure 3A). Previously, we demonstrated that cisplatin treatment reduced the mRNA levels of METTL14 and FTO in HeLa cells by 3.3- and 6.6-fold, respectively (Alasar et al., 2022). To investigate if a similar expression pattern is observed in breast and lung cancer cells, we performed qPCR analyses with total RNAs isolated from cisplatin-treated MCF7 and A549 cells. Similarly, cisplatin treatment led to a significant reduction in the transcript abundance of METTL14 by 1.5-fold (p < 0.05) and 1.7-fold (p < 0.05) in MCF7 and A549 cells, respectively. FTO was downregulated by 9.2-fold (p < 0.0001) in cisplatin-treated MCF7 and 7.7-fold (p < 0.01) in cisplatin-treated A549 cells. Additionally, RBM15 expression was downregulated by 1.4-fold (p < 0.05) in cisplatin-treated A549 cells (Figure 3B). Next, we examined the transcript abundance of m^6^A readers in HeLa, MCF7, and A549 cells upon cisplatin treatment. Interestingly, readers displayed distinct expression patterns, although similar expression levels of writers and erasers were observed in all cell lines. In cisplatin-treated HeLa cells, YTHDF1 and YTHDF3 were upregulated by 2.6-fold (p < 0.01) and 1.3-fold (p < 0.05), respectively, while YTHDC2, IGF2BP2, IGF2BP3, and HNRNPA2B1 were downregulated by 2.1-fold (p < 0.01), 1.3-fold (p < 0.0001), 2.9-fold (p < 0.01), and 1.5-fold (p < 0.05), respectively (Figure 3C). In MCF7 cells, cisplatin treatment resulted in a reduction in transcript levels of IGF2BP2, IGF2BP3, HNRNPA2B1, and HNRNPG by 1.7-fold (p < 0.05), 7.1-fold (p < 0.0001), 1.4-fold (p < 0.05), and 1.9-fold (p < 0.05), respectively (Figure 3C). Similarly, in cisplatin-treated A549 cells, the expression of YTHDC2, IGF2BP1, IGF2BP2, IGF2BP3, HNRNPA2B1, and HNRNPC was downregulated by 2.3-fold (p < 0.05), 2.2-fold (p < 0.05), 3.3-fold (p < 0.001), 4.7-fold (p < 0.01), 2.3-fold (p < 0.05), and 1.9-fold (p < 0.001), respectively (Figure 3C). Collectively, these analyses demonstrate that cisplatin treatment leads to a reduction in the abundance of most m^6^A RNA modification regulators under our experimental conditions.

### 3.4. Expressions of m^6^A regulators in TNF-α-treated cancer cells

**Figure 4:**
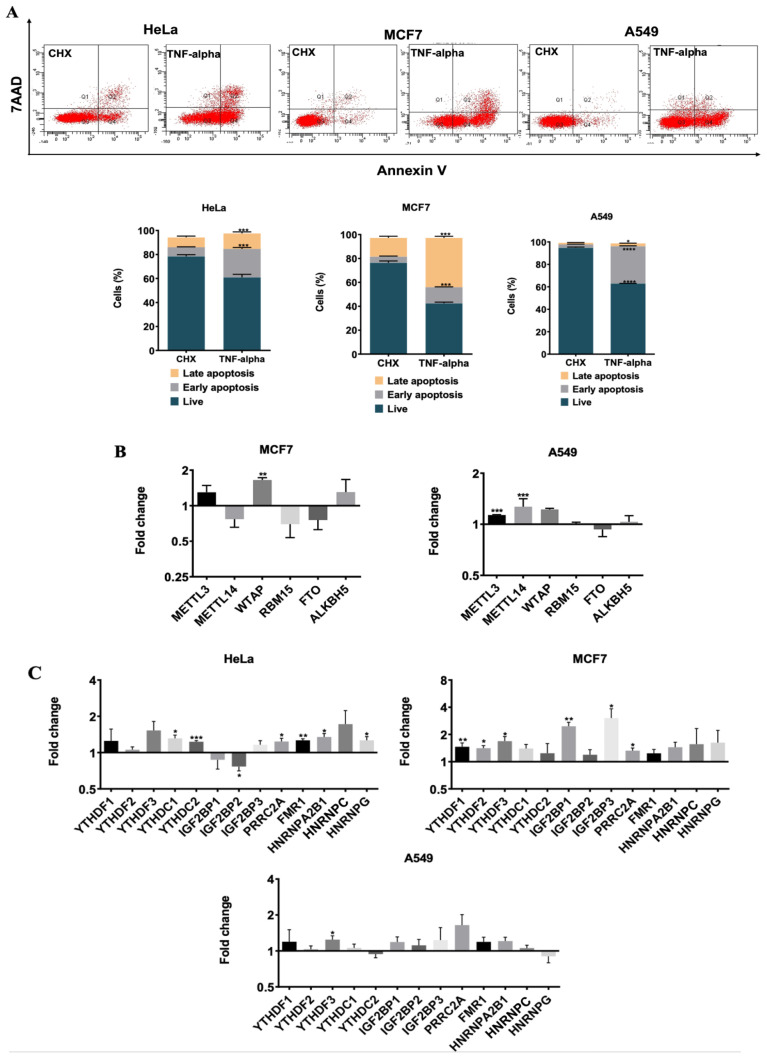
Gene expression analysis in TNF-α-induced apoptotic HeLa, MCF7, and A549 cells**. (A)** Less metastatic HeLa, MCF7, and A549 cells were treated with 75 ng/mL TNF-α in 5 μg/mL CHX, 10 ng/mL TNF-α in 5 μg/mL CHX and 20 ng/mL TNF-α in 10 μg/mL CHX for 24 h, respectively. CHX was used as a control. **(B)** Gene expression levels of m^6^A writers and erasers in TNF-α induced apoptotic MCF7 (left) and A549 (right) cells. **(C)** Gene expression levels of m^6^A readers in HeLa, MCF7, and A549 cells under TNF-α-treated condition. All qPCR samples were normalized with GAPDH housekeeping gene. Two-tailed Student’s *t*-test was performed to determine the statistical significance among groups. n =3 biological replicates. Data presented as mean ± SD, *: p ≤ 0.05, **: p ≤ 0.01, ***: p ≤ 0.001****: p ≤ 0.0001.

Although cisplatin induces apoptosis through the intrinsic pathway, TNF-α primarily triggers apoptosis by activating the extrinsic pathway (Akçaöz Alasar et al., 2024). We hypothesized that the abundance of m^6^A RNA regulators should be pathway-specific. To address the effects of TNF-α treatment on the abundance of m^6^A RNA regulators, we treated HeLa, MCF7, and A549 cells with TNF-α at concentrations of 75 ng/mL, 10 ng/mL, and 20 ng/mL, respectively, with CHX at concentrations of 10 μg/mL, 5 μg/mL, and 10 μg/mL, respectively. TNF-α reduced the rate of live cells to 60% while causing an apoptosis rate of 23.6% in HeLa cells (Figure 4A). TNF-α induced early apoptosis rates of 14% and 33% in MCF7 and A549 cells, respectively (Figure 4A). We previously reported that, among all readers tested, only the abundance of WTAP transcript was upregulated by 2.8-fold upon TNF-α treatment of HeLa cells (Alasar et al., 2022). We further explored the abundance of m^6^A regulators in TNF-α-treated MCF7 and A549 cells. However, we did not observe any dramatic difference in the expression of m^6^A regulators, except for WTAP (1.6-fold, p < 0.01) in MCF7 cells. The effect of TNF-α on the abundance of METTL3 and METTL14 transcripts was quite marginal, with a 1.2-fold elevation (p < 0.001) (Figure 4B). It appears that METTL14 and FTO downregulations are specific to CP-induced apoptosis (Figure 3B) rather than TNF-α (Figure 4B). Moreover, we performed qPCR analyses to investigate the transcript levels of readers under TNF-α-induced apoptotic conditions. We did not detect any discernible change in the amount of YTHDC1, YTHDC2, PRR2CA, FMR1, HNRNPA2B1, and HNRPNG in TNF-α-treated HeLa cells (Figure 4C). TNF-α treatment of MCF7 cells led to a 2.4-fold (p < 0.01) and 3-fold (p < 0.05) increase in the IGF2BP1 and IGF2BP3 transcript levels, respectively (Figure 4C). There was no apparent difference in the levels of readers in A549 cells (Figure 4C). In summary, our findings showed that the expression of m^6^A regulators tends to decrease under CP-induced apoptotic conditions, whereas TNF-α treatment promotes upregulation.

## 4. Discussion

The most prevalent cancer types, including breast, colorectal, lung, and cervical cancers, have been identified by the World Health Organization. With its diverse impact on tumorigenesis, m^6^A RNA methylation has recently emerged as a significant contributor to this process. In this study, we present expression profiles of m^6^A methylation machinery in breast, colorectal, lung, and cervical cancer cell lines. CESC, BRCA, LUAD, and COAD cell lines express lower amounts of METTL3 and FTO transcripts compared to their healthy counterparts, while WTAP and ALKBH5 expressions differ by cancer type (Figure 1). However, it is crucial to consider the genomic differences among cell lines when interpreting differences in the expression of RNA m^6^A modifiers. Previously, we reported that cisplatin-mediated apoptosis is modulated by the METTL3-PMAIP1 axis in HeLa cells (Alasar et al., 2022). In this study, our analysis of cisplatin-mediated apoptosis reveals a significant reduction in METTL14 and FTO in all cell types (Figure 3). METTL14 has been identified as an oncogene in pancreatic cancer, as its depletion increases susceptibility to cisplatin-induced apoptosis in PANC-1 and CFPAC-1 cells (Kong et al., 2020). METT14 may act as an intermediate component in cisplatin-mediated apoptosis due to its decrease following cisplatin treatment, as depicted in Figure 3. Additionally, there are reports suggesting the protective role of FTO against cisplatin-induced cytotoxicity. For example, cisplatin treatment decreases FTO expression, and downregulation of FTO enhances m^6^A methylation level and sensitizes cells to cisplatin (Zhou et al., 2019). These findings suggest that differential m^6^A methylation via dysregulation of METTL14 or FTO may aggravate cisplatin-mediated apoptosis. However, further studies are required to elucidate the common targets of cisplatin across cancer types.

In our study, TNF-α treatment did not reveal any common expression differences for writer and eraser genes (Figure 4). This observation suggests that the reduction of METTL14 and FTO may be specific to the CP-mediated intrinsic pathway. Interestingly, cisplatin treatment dramatically reduced the expression levels of specific readers, IGF2BP2 and IGF2BP3, compared to TNF-α treatment (Figures 3 and 4). These findings align with previously reported studies. Notably, ncRNA-IGF2BP2 complexes play a role in cancer pathogenesis (Huang et al., 2018; Ma et al., 2021). Moreover, IGF2BP2 promotes the growth and metastasis of cervical cancer cells (Hu et al., 2022). Upregulation of IGF2BP2 by a miRNA-lncRNA interaction has been reported to result in increased apoptosis and decreased proliferation. Thus, a miRNA-lncRNA-IGF2BP2 axis renders cervical cancer cells resistant to cisplatin (Wu et al., 2022). A similar observation has been reported regarding IGF2BP2 increasing cisplatin resistance in colorectal cancer (Xia et al., 2022). However, our results indicate that IGF2BP2 expression is decreased specifically upon cisplatin-induced apoptosis, suggesting a potential negative correlation between cisplatin and IGF2BP2. Additionally, the abundance of IGF2BP3 transcript was reduced upon cisplatin treatment (Figure 3). IGF2BP3 has been associated with gastric cancer progression, with high expression in four gastric cancer subtypes indicating its potential role in promoting cell growth and invasion (Zhou et al., 2017). Yang et al. (2023) reported the oncogenic and poor prognostic properties of IGF2BP3 and provided evidence that downregulation of IGF2BP3 leads to enhanced apoptosis. Moreover, IGF2BP3 has been linked to cisplatin resistance in laryngeal cancer (Yang et al., 2023), providing further evidence for the potential role of IGF2BP3 in cisplatin-mediated apoptosis.

In conclusion, while further experiments are needed to fully understand the molecular differences between the intrinsic and extrinsic apoptotic pathways, our study highlights differences in the abundance of the m^6^A methylation machinery within these pathways. Given that expression differences in m^6^A regulators are expected to affect the genome-wide m^6^A methylation profile, investigating the m^6^A RNA methylome under intrinsic and extrinsic apoptotic conditions becomes crucial. Moreover, additional experiments are warranted to elucidate the significance of opposing expression of METTL14, FTO, IGF2BP2, and IGF2BP3 transcripts in cisplatin- and TNF-α-mediated apoptotic pathways.

## Figures and Tables

**Table:** List of primers used in qPCR analysis.

Genes	Forward 5′-3′	Reverse 5′-3′
METTL3	AGATGGGGTAGAAAGCCTCCT	TGGTCAGCATAGGTTACAAGAGT
METTL14	GAGTGTGTTTACGAAAATGGGGT	CCGTCTGTGCTACGCTTCA
WTAP	TTGTAATGCGACTAGCAACCAA	GCTGGGTCTACCATTGTTGATCT
RBM15	AAGATGGCGGCGTGCGGTTCCGCTGTG	AAGTTCACAAAGGCTACCCGCTCATCC
FTO	CTTCACCAAGGAGACTGCTATTTC	CAAGGTTCCTGTTGAGCACTCTG
ALKBH5	TCCAGTTCAAGCCTATTCG	CATCTAATCTTGTCTTCCTGAG
YTHDF1	TAAGGAAATCCAATGGACGG	TTTGAGCCCTACCTTACTGGA
YTHDF2	CCTTAGGTGGAGCCATGATTG	TCTGTGCTACCCAACTTCAGT
YTHDF3	TGACAACAAACCGGTTACCA	TGTTTCTATTTCTCTCCCTACGC
YTHDC1	TCAGGAGTTCGCCGAGATGTGT	AGGATGGTGTGGAGGTTGTTCC
YTHDC2	GTGTCTGGACCCCATCCTTA	CCCATCACTTCGTGCTTTTT
IGF2BP1	TAGTACCAAGAGACCAGACCC	GATTTCTGCCCGTTGTTGTC
IGF2BP2	ATCGTCAGAATTATCGGGCA	GCGTTTGGTCTCATTCTGTC
IGF2BP3	AGACACCTGATGAGAATGACC	GTTTCCTGAGCCTTTACTTCC
PRRC2A	AGGGCAAGTCCTTAGAGATCC	TTCAGGCTTGGAAGGTTGGC
FMR1	CAGGGCTGAAGAGAAGATGG	ACAGGAGGTGGGAATCTGA
HNRNPA2B1	AGCTTTGAAACCACAGAAGAA	TTGATCTTTTGCTTGCAGGA
HNRNPC	TAAGGAAATCCAATGGACGG	TTTGAGCCCTACCTTACTGGA
HNRNPG	TAAGGAAATCCAATGGACGG	TTTGAGCCCTACCTTACTGGA
GAPDH	ACTCCTCCACCTTTGACGC	GCTGTAGCCAAATTCGTTGTC
